# Association of Poor Sleep Efficiency With Decreased Executive Function and Impaired Episodic Memory in Older Adults

**DOI:** 10.7759/cureus.90061

**Published:** 2025-08-14

**Authors:** Akansha Singh, Rishabh Soni, Gauri Shankar Kaloiya, Avinash Chakrawarty, Manjari Tripathi, Ratna Sharma, Nasreen Akhtar

**Affiliations:** 1 Department of Physiology, All India Institute of Medical Sciences, New Delhi, IND; 2 Department of Psychiatry, National Drug Dependence Treatment Centre, All India Institute of Medical Sciences, New Delhi, IND; 3 Department of Geriatric Medicine, All India Institute of Medical Sciences, New Delhi, IND; 4 Department of Neurology, All India Institute of Medical Sciences, New Delhi, IND

**Keywords:** aging, cognition, executive function, icmr-nctb, memory, psqi, sleep quality

## Abstract

Background: Sleep characteristics influence both physical and psychological health. While previous studies have reported links between sleep quality and cognitive impairment in older adults, findings remain inconsistent, and evidence from the Indian population using culturally validated cognitive tools is scarce.

Objective: This study aims to examine the relationship between subjective sleep quality and cognitive performance in Indian older adults using the Pittsburgh Sleep Quality Index (PSQI) and the Indian Council of Medical Research-Neurocognitive Toolbox (ICMR-NCTB).

Methods: In this cross-sectional study, 67 individuals aged 50-80 years completed the PSQI and the Hindi version of the ICMR-NCTB. Participants were categorized as good (PSQI ≤ 5) or poor (PSQI > 5) sleepers. Cognitive domains assessed included global cognition, language, attention, visuospatial ability, executive function, and episodic memory. Group comparisons and correlation analyses were performed to explore associations between sleep quality and cognitive outcomes.

Results: Sleep latency and sleep efficiency were significantly associated with cognitive performance. Longer sleep latency correlated with poorer executive function (Trail Making Test (TMT)-B: r = 0.347, p = 0.006; TMT-(B-A): r = 0.289, p = 0.023) and reduced performance in verbal learning (Verbal Learning Test (VLT) delayed recall: r = -0.183, p = 0.141; delayed recognition: r = -0.284, p = 0.02). Higher sleep efficiency was linked to better executive function and episodic memory. Poor sleepers scored significantly lower on global cognition (Montreal Cognitive Assessment (MoCA), p = 0.047), executive function (TMT-B*,* *p* = 0.029), verbal fluency (Phonemic Fluency Task (PFT), p = 0.031), and delayed recall (VLT, p = 0.045) compared to good sleepers. Education was positively associated with most cognitive scores but not with sleep quality.

Conclusion: Prolonged sleep latency and poor sleep efficiency were associated with deficits in executive function and memory among older adults. These findings underscore the importance of sleep quality in cognitive aging, and the use of a culturally adapted cognitive tool enhances their applicability in low-literacy, non-Western settings.

## Introduction

Sleep is a physiological process essential for physical restoration and optimal brain function [1]. It supports attention, executive processing, memory consolidation, and learning, processes that depend on coordinated neural activity during slow-wave and rapid eye movement (REM) sleep [2]. With increasing age, changes in sleep architecture, such as reduced slow-wave sleep, greater fragmentation, and lower efficiency, are common and often parallel declines in cognitive abilities, particularly in attention, executive function, and episodic memory [3]. 

Sleep complaints are highly prevalent among older adults but often under-recognized. These disturbances frequently coincide with declines in processing speed, attention, executive function, and episodic memory [4,5]. Epidemiological and clinical studies often use validated subjective tools such as the Pittsburgh Sleep Quality Index (PSQI) to assess sleep quality in aging populations. While several studies have linked poor sleep quality with impairments in executive functioning, episodic memory, and global cognition [6,7], findings remain inconsistent-likely due to methodological differences and variations in study populations [8,9]. Some studies found no significant relationship when using broad screening measures like the Mini-Mental State Examination (MMSE) [4], whereas others detected deficits when domain-specific cognitive assessments were applied [10].

In India, research on sleep-cognition associations is limited, particularly studies employing culturally validated tools [11,12]. The Indian Council of Medical Research-Neurocognitive Toolbox (ICMR-NCTB) provides a standardized, linguistically adapted battery suitable for diverse and low-literacy populations [13]. Given India’s rapidly aging demographic, identifying modifiable factors such as sleep that may influence cognitive aging is increasingly important.

This study examined the association between subjective sleep quality, measured by the PSQI, and cognitive performance in Indian older adults using the ICMR-NCTB. We evaluated both overall cognition and specific domains, language, attention, visuospatial ability, executive function, and episodic memory, to better understand how different aspects of sleep relate to cognitive abilities in this population.

## Materials and methods

Study design and participants 

**Figure 1:**
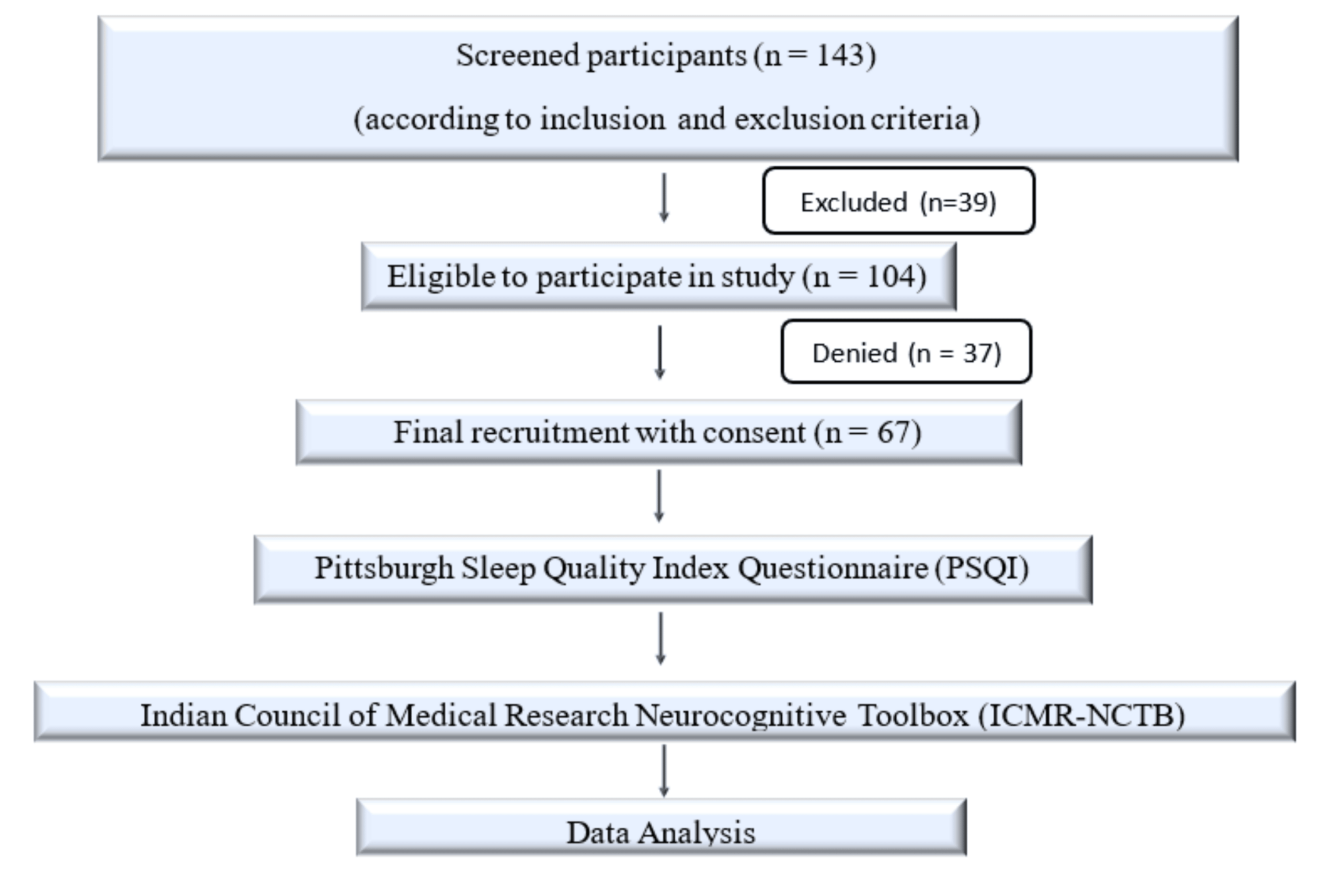
Participant recruitment flow diagram

This cross-sectional observational study, part of the SOLACE study [14], was conducted between April 2022 and March 2024. A total of 67 participants (both men and women) aged 50-80 years were recruited from the Department of Geriatrics outpatient clinic at the All India Institute of Medical Sciences, New Delhi, and from residential communities in South Delhi. Exclusion criteria included non-ambulatory status, a diagnosis of major depressive disorder or generalized anxiety disorder, any history of psychiatric illness, or current use of psychotropic medications. Written informed consent was obtained from all participants prior to enrolment. The participant recruitment flow is shown in Figure 1. The study was approved by the Institutional Ethics Committee (IECPG-349/20.07.2023, RT-21/24.08.2023). 

Assessment of sleep quality 

Subjective sleep quality was assessed using the PSQI [15], a self-administered questionnaire that evaluates sleep quality and disturbances over the past month. It comprises 19 items grouped into seven components: subjective sleep quality, sleep latency, sleep duration, habitual sleep efficiency, sleep disturbances, use of sleep medications, and daytime dysfunction. Each component is scored from 0 to 3, and the scores are summed to yield a global score ranging from 0 to 21, with higher scores indicating poorer sleep quality. A global score of ≤5 was considered good sleep quality. For the sleep efficiency and overall sleep quality components, lower values indicated better sleep. The questionnaire takes about 5-7 minutes to complete.

Cognitive assessment 

Cognitive function was assessed using the ICMR-NCTB, a validated paper-based battery designed to evaluate multiple cognitive domains in the Indian population [13]. It measures global cognition, attention and executive function, episodic memory, language, and visuospatial skills, and is available in five Indian languages: Hindi, Bengali, Telugu, Kannada, and Malayalam. The Hindi version was used in this study.

Global cognition was assessed using the Montreal Cognitive Assessment (MoCA), which covers attention, memory, executive function, language, visuoconstruction, conceptual thinking, calculations, and orientation. Specific domains were evaluated using the following: Trail Making Test (TMT) for attention, speed, and cognitive flexibility; Category Fluency Task (CFT) for semantic memory, executive function, and language; Phonemic Fluency Task (PFT) for executive processing and word retrieval; Verbal Learning Test (VLT) for episodic memory, adapted from the "Consortium to Establish a Registry for Alzheimer’s Disease" (CERAD) 10-word list, administered over three immediate recall trials, followed by a delayed recall after 20 minutes to test immediate recall, delayed recall, and recognition [16]. Additional cognitive tests included the Test des Neuf Images du 93 (TNI-93), a brief episodic memory test suitable for low-literacy individuals; Picture Naming Test (PNT) for language and semantic memory, by asking participants to name 30 line-drawn objects, with category and phonemic cues provided if necessary; and Modified Taylor Complex Figure (MTCF) test for visuoconstruction, visual memory, and learning (copy, immediate recall, and delayed recall). The ICMR-NCTB battery, designed to screen for early cognitive impairment and dementia, is especially appropriate in the Indian context due to its cultural and linguistic adaptability.

Detailed administration and scoring procedures followed the ICMR-NCTB manual [13]. MoCA was scored out of 30, with higher scores indicating better cognition. TMT was scored by completion time and B-A difference; CFT and PFT by the number of correct words; VLT by immediate recall (three trials), delayed recall, and recognition scores; TNI-93 by correct recall; PNT by correct naming (with or without cues); and MTCF by accuracy across copy and recall conditions. Scores below the 15th percentile, adjusted for age and education as per ICMR-NCTB norms, were considered indicative of cognitive impairment.

Statistical analysis 

Data were analyzed using GraphPad Prism version 10.4.0 (Dotmatics, Boston, MA, USA; www.graphpad.com). Post hoc sample size calculation was performed for a one-tailed t-test comparing means between two independent groups. A medium effect size (Cohen’s d = 0.66) was estimated from the observed difference in CFT vegetable scores between groups. With a type I error rate (α) of 0.05, the achieved statistical power was 84.7% (1 − β = 0.847), corresponding to a minimum sample size requirement of 65 participants. This calculation was performed using G*Power version 3.1.9.7 (Universität Düsseldorf, Düsseldorf, Germany) [17]. To account for potential dropouts or missing data, a slightly larger sample was targeted. Group-wise comparisons were conducted using unpaired two-tailed Student’s t-test or the Mann-Whitney U test, as appropriate. Correlations were assessed using Pearson’s or Spearman’s correlation coefficients, depending on data normality. A p-value < 0.05 was considered statistically significant. Variables showing significant correlations were further analyzed using linear regression.

## Results

Participant characteristics 

The mean age of the study population was 65 ± 7.02 years, comprising 54 men and 13 women, with a mean BMI of 27.9 ± 5.4 kg/m². The median height was 165 cm, and the mean weight was 73.3 ± 14.10 kg. Among the participants, good sleepers (n = 34) had a mean age of 64.21 ± 7.3 years, whereas poor sleepers (n = 33) had a mean age of 65.82 ± 6.69 years. Detailed baseline characteristics are summarized in Table 1.

**Table 1:** Baseline demographic characteristics of participants Values are expressed as mean ± standard deviation for normally distributed data, or as median (Q3-Q1) for non-parametric data. For comparison between two groups, the unpaired two-tailed Student’s t-test was used for parametric data and the Mann-Whitney U test for non-parametric data. p < 0.05 was considered statistically significant. M: male, F: female, PSQI: Pittsburgh Sleep Quality Index.

Parameters	Good sleepers (n = 34)	Poor sleepers (n = 33)	p-value
Age	64.21 ± 7.3	65.82 ± 6.69	0.351
Sex	(M: 26, F: 8)	(M: 28, F: 5)	
Height (cm)	163.88 ± 8.06	163.88 ± 8.06	0.952
Weight (kg)	75.04 ± 15.62	71.57 ± 12.34	0.317
BMI	27.5 (31.18-24.74)	27 (30.86-23.5)	0.475
PSQI score	3.00 (4-2)	7.00 (10-6)	<0.0001
Waist-to-hip ratio	0.96 ± 0.08	1.00 ± 0.07	0.082
Years of education	12 (15.00-10.00)	10 (13.5-7.5)	0.121
Cardiovascular diseases	10	8	
Diabetes type II	10	12	
Hypertension	19	21	

Correlates of cognition in the whole group****

In the whole group, the global PSQI score was positively correlated with TMT-B performance time (r = 0.28, p = 0.02) (Figure 1A) and negatively correlated with VLT delayed recall (r = -0.254, p = 0.037) and VLT delayed recognition (r = -0.236, p = 0.054) (Figure 1B). Non-significant negative correlations were also observed between the global PSQI score and MTCF delayed recall (r = -0.133, p = 0.305), MTCF immediate recall (r = -0.098, p = 0.440), MTCF copy (r = -0.141, p = 0.269), and PNT total score (r = -0.081, p = 0.519), as well as a non-significant positive correlation with TMT-A (r = -0.202, p = 0.105). Sleep latency was positively correlated with executive functioning as measured by TMT-B (r = 0.347, p = 0.006) (Figure 1C), and showed negative correlations with VLT delayed recall (r = -0.183, p = 0.141), VLT delayed recognition (r = -0.284, p = 0.02) (Figure 1D), and TNI-93 total recall (r = -0.286, p = 0.056). It was also positively correlated with TMT-(B-A) (r = 0.289, p = 0.023) and negatively correlated with total PFT score (r = -0.249, p = 0.045). In addition, sleep latency showed a significant negative correlation with MTCF delayed recall (r = -0.293, p = 0.023) and non-significant negative correlations with MTCF copy (r = -0.183, p = 0.154) and a non-significant positive correlation with PNT total score (r = 0.067, p = 0.597) and TMT-A (r = 0.228, p = 0.069). Sleep efficiency showed a negative association with TMT-B performance time (r = -0.451, p = 0.0002) (Figure 1E) and positive associations with multiple cognitive measures, including CFT vegetables (r = 0.382, p = 0.001), CFT food items (r = 0.286, p = 0.018), VLT delayed recognition (r = 0.369, p = 0.002) (Figure 1F), and the PNT (r = 0.267, p = 0.031). Higher education correlated with better global cognition (MoCA score) (r = 0.629, p < 0.001), PFT (r = 0.522, p < 0.001), and CFT vegetables (r = 0.255, p = 0.036) and showed negative associations with executive dysfunction (TMT-(B-A); r = -0.437, p < 0.001), CFT animals (r = -0.320, p = 0.008), and CFT food items (r = -0.278, p = 0.022). 

**Figure 2:**
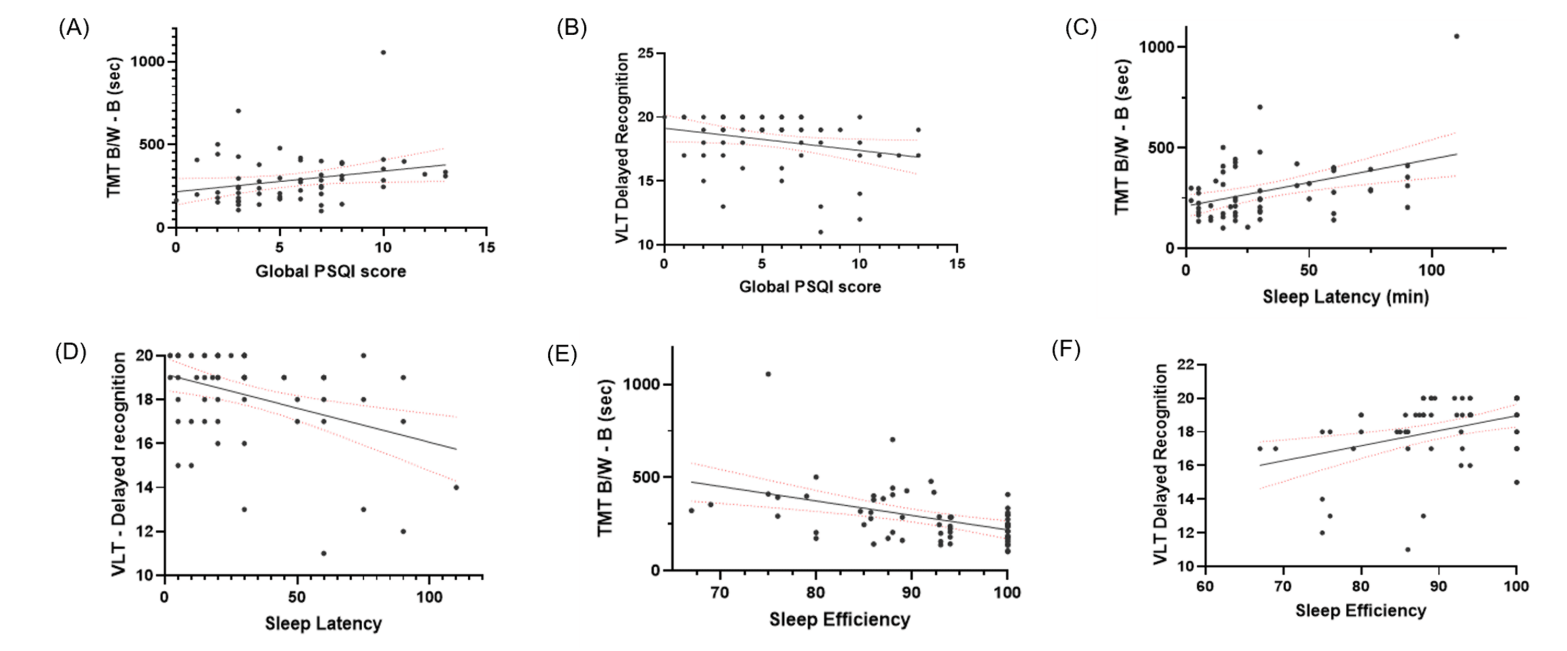
Linear regression plots showing associations between sleep parameters and cognitive performance (A) PSQI global score vs TMT-B (β = 12.42, SE = 6.24, F = 3.953, p = 0.051, 95% CI: -0.075 to 24.91). (B) PSQI global score vs VLT delayed recognition (β = -0.174, SE = 0.08, F = 4.409, p = 0.039, 95% CI: -0.34 to -0.008). (C) Sleep latency vs TMT-B (β = 2.342, SE = 0.65, F = 12.62, p = 0.0007, 95% CI: 1.02-3.66). (D) Sleep latency vs VLT delayed recognition (β = -0.031, SE = 0.008, F = 12.05, p = 0.0009, 95% CI: -0.048 to -0.013). (E) Sleep efficiency vs TMT-B (β = -7.777, SE = 1.98, F = 15.33, p = 0.0002, 95% CI: -11.75 to -3.805). (F) Sleep efficiency vs VLT delayed recognition (β = 0.089, SE = 0.02, F = 10.75, p = 0.001, 95% CI: 0.034-0.143). TMT-B: Trail Making Test-B, VLT: Verbal Learning Test.

Correlation of sleep and cognition in the good sleepers 

Among good sleepers (PSQI ≤ 5), sleep efficiency was positively associated with performance on the PNT (r = 0.087, p = 0.629) and MTCF delayed recall (r = 0.447, p =0.013), indicating a relationship between more efficient sleep and higher cognitive performance. Daytime dysfunction scores were negatively correlated with PNT (r = -0.353, p = 0.043). Years of education were associated with MoCA (r = 0.515, p = 0.002), MTCF copy (r = 0.596, p = 0.001), MTCF immediate recall (r = 0.530, p = 0.002), and MTCF delayed recall (r = 0.553, p = 0.002). It also correlated with CFT animals (r = 0.444, p = 0.008), CFT vegetables (r = 0.441, p = 0.016), PFT (r = 0.614, p < 0.001), and PNT (r = 0.461, p = 0.007), and negatively with TMT-(B-A) (r = -0.465, p = 0.007). Non-significant positive correlations were observed between the global PSQI score and MTCF delayed recall (r = 0.327, p = 0.077), MTCF copy (r = 0.184, p = 0.312), and PNT (r = 0.169, p = 0.347), while a non-significant negative correlation was seen with TMT-A (r = -0.160, p = 0.356). Similarly, sleep latency showed non-significant negative correlations with MTCF delayed recall (r = -0.042, p = 0.822), MTCF immediate recall (r = -0.117, p = 0.521), and TMT-A (r = -0.069, p = 0.697), as well as a non-significant positive correlation with MTCF copy (r = 0.117, p = 0.521). Sleep efficiency showed a non-significant positive correlation with MTCF copy (r = 0.076, p = 0.675). 

Correlation of sleep and cognition in the poor sleepers 

In the poor sleeper group (PSQI > 5), higher overall sleep quality scores (indicating poorer sleep) were associated with lower cognitive scores. Negative correlations were observed with MTCF delayed recall (r = -0.369, p = 0.041), MTCF immediate recall (r = -0.410, p = 0.022), and MoCA (r = -0.407, p = 0.019). Sleep latency showed a positive correlation with TMT-B (r = 0.452, p = 0.012) and a negative correlation with total PFT score (r = -0.387, p = 0.028). Sleep efficiency score also showed a positive correlation with VLT delayed recognition (r = 0.421, p = 0.014), and global PSQI score was negatively correlated with the same measure (r = -0.318, p = 0.07). Years of education correlated positively with MoCA (r = 0.685, p < 0.001), MTCF copy (r = 0.801, p < 0.001), MTCF immediate recall (r = 0.557, p = 0.001), and MTCF delayed recall (r = 0.532, p = 0.002). Years of education received was not significantly correlated with any of the sleep parameters. Additional non-significant correlations included negative associations between the global PSQI score and MTCF delayed recall (r = -0.237, p = 0.197), MTCF copy (r = -0.070, p = 0.706), MTCF immediate recall (r = -0.251, p = 0.172), and PNT total score (r = -0.209, p = 0.249), as well as a positive correlation with TMT-A (r = 0.305, p = 0.095). Sleep latency also showed non-significant negative correlations with MTCF delayed recall (r = -0.336, p = 0.064) and MTCF copy (r = -0.175, p = 0.345), and a non-significant positive correlation with TMT-A (r = 0.337, p = 0.063). Sleep efficiency showed a non-significant positive correlation with MTCF copy (r = 0.246, p = 0.181). 

Comparison of cognition and sleep quality between good and poor sleepers

Comparisons between good and poor sleepers were conducted for MoCA, TMT-A, and TMT-B, CFT, PFT, and VLT (assessing immediate recall, delayed recall, and delayed recognition), as well as PNT and MTCF (copy, immediate recall, and delayed recall). The results of these comparisons are presented in Table 2. Component-level PSQI score comparisons between good and poor sleepers are shown in Table 3.

**Table 2:** Comparison of cognitive test scores between good sleepers (GS) and poor sleepers (PS) Cognitive performance was compared between participants categorized as good sleepers (GS) and poor sleepers (PS) based on their PSQI scores. For each cognitive test, results are expressed as mean ± standard deviation for parametric data, or as median (Q3-Q1) for non-parametric data. MoCA: a global cognition screening tool scored out of 30 points. TMT-A: Time in seconds to complete the test A. TMT-B: Time in seconds to complete test B. TMT-(B-A): Time in seconds to complete TMT-B minus TMT-A. CFT: Total number of vegetable/animal/food names generated in one minute. PFT total: Sum of correct words generated across three letters (Pa, Ka, Ma). VLT: Number of correctly recalled/recognized target words immediately and after a delay over three learning trials. Test des Neuf Images du 93 (TNI-93) total recall: Number of pictures correctly recalled from a set of nine images. PNT: Name of 30 pictures correctly told. Modified Taylor Complex Figure (MTCF): Total points scored from copying, immediate recall, and delayed recall of the Modified Taylor Complex Figure. Higher scores in all the above tests denote better cognitive functions, except TMT-A, TMT-B, TMT-(B-A), where a higher score denotes poorer executive function. For comparison between two groups, the unpaired two-tailed Student’s t-test was used for parametric data and the Mann-Whitney U test for non-parametric data. *p < 0.05 was considered significant. PSQI: Pittsburgh Sleep Quality Index.

Cognitive domain	Test name	Good sleepers (n = 34)	Poor sleepers (n = 33)	p-value
Global cognition	Montreal Cognitive Assessment (MoCA)	26 (27-23)	23 (25-22)	0.047*
Executive function/attention	Trail Making Test A (TMT-A) (sec)	72 (121-61)	100 (1275-80)	0.053
	Trail Making Test B (TMT-B) (sec)	208.5 (298.5-167)	290.5 (378.8-245)	0.029*
	Trail Making Test (B-A) (TMT-(B-A)) (sec)	136.50 (214.00-98.75)	195.00 (228.50-156.00)	0.087
	Category Fluency Test (CFT) vegetable	14.71 ± 3.42	12.48 ± 3.33	0.009*
	Category Fluency Test (CFT) animal	14.47 ± 4.98	13.18 ± 3.82	0.239
	Category Fluency Test (CFT) food	12.09 ± 3.26	10.24 ± 2.75	0.015*
	Phonemic Fluency Total (PFT)	27.97 ± 12.48	22.06 ± 8.94	0.031*
Episodic memory	Verbal Learning Test (VLT) immediate recall	17.21 ± 3.44	15.79 ± 3.41	0.096
	Verbal Learning Test (VLT) delayed recall	5 (7-5)	4 (6-4)	0.045*
	Verbal Learning Test (VLT) delayed recognition	19 (20-18)	19 (19-17)	0.114
	TNI-93 total recall	9(9-9)	9 (9-8)	0.377
	MTCF immediate recall	16.78 ± 6.56	14.71 ± 8.28	p = 0.274
	MTCF delayed recall	16.28 ± 7.01	13.81 ± 8.55	p = 0.222
Language	Picture Naming Test (PNT)	89 (90-84)	87(90-84)	p = 0.451
Visuospatial and constructive ability	MTCF copy	35 (36–30)	35(36–25)	p = 0.157

**Table 3:** Comparison of PSQI component scores between good and poor sleepers Results are presented as mean ± standard deviation for both normally distributed and non-parametric data to allow ease of comparison. Subjective sleep quality, sleep disturbance, and daytime dysfunction scores were rated on a 0-3 scale, with 0 indicating the best sleep quality and 3 the worst. Sleep efficiency was calculated as (hours slept ÷ hours in bed) × 100%. N denotes the number of participants taking sleep medications. Group comparisons were performed using an unpaired two-tailed Student’s t-test for parametric data and the Mann-Whitney U test for non-parametric data. *p < 0.05 was considered statistically significant. PSQI: Pittsburgh Sleep Quality Index.

PSQI parameter	Good sleeper (n = 34)	Poor sleeper (n = 33)	p-value
Subjective sleep quality (0-3 rating)	0.52 ± 0.66	1.51 ± 0.75	<0.0001
Sleep latency (min)	18.94 ± 14.02	45.67 ± 29.37	<0.0001
Sleep duration (min)	430.6 ± 46.05	385.5 ± 66.29	0.0026
Sleep efficiency (%)	94.02 ± 6.59	87.93 ± 9.64	0.0066
Sleep disturbance score (0-3 rating)	1.14 ± 0.50	1.51 ± 0.56	0.0023
Use of sleep medication (n)	0	5	<0.0001
Daytime dysfunction score (0-3 rating)	0.44 ± 0.74	1.0 ± 1.0	0.0109

## Discussion

This study examined the relationship between self-reported sleep quality and cognitive function in older adults using the PSQI and ICMR-NCTB. Longer sleep latency and poorer sleep efficiency were associated with poorer executive function and memory, particularly in delayed recall and delayed recognition tasks. Poor sleepers also showed significantly lower global cognition and visuospatial memory. While education was a strong predictor of cognitive performance, it was not related to sleep quality. 

Better sleep quality correlated positively with attention and executive function and negatively with the verbal component of episodic memory, consistent with previous reports of similar associations between sleep quality and memory performance [18]. However, several measures, such as immediate recall, category fluency, phonemic fluency, language (PNT), and visuospatial ability (MTCF copy), showed no significant association with the global PSQI score. These null findings align with earlier studies [19] suggesting that some cognitive domains may remain relatively preserved despite poor subjective sleep quality. 

Prolonged sleep latency is a recognized marker of poor sleep quality and an early sign of cognitive vulnerability in older adults [20]. In this study, longer sleep latency was linked to poorer performance in executive function and delayed verbal memory. However, in both good and poor sleepers, sleep latency did not significantly correlate with certain visuospatial measures (MTCF copy, MTCF immediate recall) or language performance (PNT), suggesting that its effects may selectively impact specific cognitive domains while sparing others. Similar domain-specific patterns have been reported in studies using both subjective questionnaires and polysomnography, where memory and executive tasks tend to be more sensitive than naming or visuoconstructive abilities to difficulties in sleep initiation. These findings align with recent research connecting sleep quality to cognitive decline. Reffi et al. [21] found that individuals with persistent poor sleep exhibited both subjective and objective disturbances, along with deficits in five of eight visuospatial and abstract reasoning tasks on the MoCA. Similarly, Verweij et al. [22] reported that people with chronic sleep problems had increased sleep latency. This delay in sleep onset can reduce slow-wave sleep, which is essential for memory and executive function. Taken together, evidence from both subjective assessments and polysomnographic recordings suggests that prolonged sleep latency may reflect early cortical dysregulation and contribute to cognitive inefficiency in aging populations.

In our study, lower sleep efficiency was linked to poorer executive performance and reduced verbal memory. Under the PSQI scoring system, lower efficiency translates to higher component scores [15], indicating a reduced ability to maintain consolidated sleep. This aligns with literature associating fragmented or insufficient sleep with impairments in prefrontal cortex-mediated functions. Jones and Harrison [23] reported that reduced sleep efficiency diminishes functional connectivity within frontal brain networks, which are critical for executive control and working memory. Similarly, a functional MRI study [24] showed that even a single night of sleep deprivation markedly reduced prefrontal activation during verbal fluency tasks, highlighting the rapid impact of sleep loss on executive function. Lower efficiency likely disrupts the continuity of NREM and REM cycles, both essential for attention shifting, verbal retrieval, and working memory. However, not all associations were significant in our sample. For instance, in the poor sleeper group, efficiency was unrelated to MTCF copy, and in the good sleeper group, its correlations with PNT and MTCF copy were small and non-significant. This suggests that certain cognitive abilities may be more resistant to sleep continuity disturbances, at least in the earlier stages of decline. Overall, our results support the idea that it is not only the quantity, but also the continuity and restorative quality of sleep, captured by sleep efficiency, that is crucial for sustaining executive, verbal memory, visuospatial memory, and language functions in older adults.

In the present study, good sleepers scored higher than poor sleepers in global cognition, attention, executive function, and the verbal aspect of episodic memory, consistent with prior reports [24,25], though some studies have found conflicting results [26]. In both groups, the global PSQI score was not significantly correlated with TMT-A, MTCF copy, or PNT, suggesting that basic psychomotor speed and certain visuospatial or naming abilities may remain preserved until sleep disruption becomes more severe. Across the entire sample, sleep quality was not significantly related to language or visuospatial domains assessed by the PNT and MTCF [18,19]. Lower sleep efficiency was associated with poorer performance on the visual learning task, particularly delayed recognition. Although recognition memory is generally more resistant to age-related decline than free recall, it can be selectively affected by disrupted sleep continuity. In good sleepers, sleep efficiency was related to PNT and MTCF delayed recall performance; however, the association with PNT was small and non-significant, suggesting that any influence of sleep continuity on naming ability may be weak or indirect without broader cognitive impairment. Subtle inefficiencies in sleep may affect word-finding ability or reflect early cognitive changes not yet evident across domains.

In poor sleepers, poorer sleep quality correlated with reduced global cognition, along with lower immediate and delayed recall scores on the MTCF, indicating impaired visuospatial memory and encoding. Nonetheless, correlations between PSQI and certain visuospatial measures (e.g., MTCF copy) were small and non-significant, suggesting that not all visuospatial components are equally sensitive to subjective sleep quality. These findings are consistent with evidence that disrupted sleep continuity and reduced efficiency impair declarative memory consolidation and executive function, processes dependent on intact slow-wave sleep and stable REM cycles [27]. Fragmented sleep has been shown to disrupt hippocampal and frontoparietal network activity, which supports visuospatial memory, attention regulation, and integrative cognitive performance, and these effects are increasingly observed in older adults with self-reported sleep disturbances. 

Education was strongly associated with better performance across cognitive tests, consistent with previous findings [28]. However, in our study, it showed no relationship with sleep quality. A likely explanation is that individuals with more years of education tend to perform better on cognitive assessments due to greater language proficiency and familiarity with structured tasks, potentially boosting scores even when underlying cognitive ability is similar to others. Zhong et al. [28] reported that education is negatively correlated with cognitive impairment, supporting its neuroprotective role. Thus, education may enhance cognitive outcomes by increasing brain reserve and adaptability, while also improving test-taking performance through better linguistic and procedural skills, factors that may inflate scores without necessarily indicating substantive differences in cognitive function. 

Several limitations should be acknowledged. First, the cross-sectional design precludes determining causal relationships between sleep characteristics and cognition. Second, sleep was assessed through self-report, which may not accurately capture objective sleep patterns, as more precise methods such as actigraphy or polysomnography were not used. Third, we did not control for potential confounders, including mood disorders, medication use, and chronic medical conditions, that could influence both sleep and cognition; adjusting for these variables would require a larger sample to maintain sufficient statistical power. The sample size itself was modest, with limited representation of women. In addition, several measures (e.g., TMT-A, MTCF copy, and PNT) showed non-significant correlations, suggesting that certain cognitive domains may be less sensitive markers of early or mild sleep-related decline. Some associations also displayed considerable variability in scatter plots, indicating weak effect sizes and warranting caution when interpreting linear trends, even if statistically significant. Larger, well-powered studies are needed to confirm these patterns and clarify the directionality of effects.

Despite these limitations, the study offers several strengths. It is among the few investigations in India to examine the sleep-cognition relationship in older adults using a culturally adapted and linguistically validated cognitive battery (ICMR-NCTB). Assessing multiple cognitive domains allowed us to identify which aspects of cognition may be selectively affected by sleep disturbances. The use of both global and component-level PSQI scores provided a more nuanced analysis of sleep variables and their potential cognitive correlates. Furthermore, the study’s applicability to low-literacy and non-Western populations enhances its public health relevance, particularly in resource-limited settings.

Future research should adopt longitudinal designs to clarify the directionality and temporal dynamics of the sleep-cognition relationship. Incorporating objective sleep assessments, such as actigraphy or polysomnography, would help validate self-reported measures. Increasing sample size and ensuring balanced gender representation would improve generalizability. Controlling for comorbid psychiatric and medical conditions will also be essential to minimize potential confounding. Intervention-based studies, such as promoting sleep hygiene, even in individuals without current deficits in domains showing non-significant associations, may enhance both sleep quality and cognitive performance. If implemented early, such approaches could serve as effective, non-pharmacological strategies to support healthy cognitive aging, especially in populations at risk for age-related decline.

## Conclusions

Prolonged sleep latency and reduced sleep efficiency were associated with impairments in executive function and episodic memory in older adults. These effects appeared domain-specific, with no impact observed on language or visuospatial skills. Using a linguistically and culturally validated cognitive tool enhances the relevance of these findings in the Indian context. Overall, the results underscore the need for further research to clarify causal pathways and to develop interventions that can help preserve cognitive health in aging populations.
